# A Survey of Koreans on Sleep Habits and Sleeping Symptoms Relating to Pillow Comfort and Support

**DOI:** 10.3390/ijerph17010302

**Published:** 2020-01-01

**Authors:** Juhyun Son, Sungwook Jung, Haseung Song, Jihee Kim, Seonghwan Bang, Sangwoo Bahn

**Affiliations:** Department of Industrial and Management Systems Engineering, Kyung Hee University, 17104 Yongin, Korea; 12thswngus@naver.com (J.S.); jungsw06310@gmail.com (S.J.); hon1415@gmail.com (H.S.); ji_hee@khu.ac.kr (J.K.); bsh7678@gmail.com (S.B.)

**Keywords:** sleep quality, sleeping position, pillow, pillow design, comfort

## Abstract

The number of people who complain of sleep disturbances is steadily increasing. An understanding of sleep-related factors is required to address sleep problems. This survey study investigated the sleep habits and sleeping symptoms relating to the comfort and support characteristics of pillows and the relationship between sleep quality and pillow design factors. The study utilized data from 332 participating Korean adults aged 20–76 years (mean age ± SD: males, 40.4 ± 15.2; females, 42.9 ± 15.4). We developed a questionnaire that evaluated sleep habits (sleep duration, bedtime, wake-up time and sleeping position); sleeping symptoms (snoring or coughing, breathing and sleepiness during waking hours) based on the Korean version of the Pittsburgh Sleep Quality Index (PSQI-K) questionnaire; and pillow-related factors (support, comfort, fatigue, height and shape) from existing pillow studies. The average sleep duration was 6.8 h, with more than half (52%) of participants sleeping in the supine position. The overall score for sleep quality was considered poor (4.84 points on a seven-point Likert scale), with some degree of sleepiness during waking hours (4.4 points on a seven-point Likert scale). Females went to bed earlier than males and were more likely to sleep in the lateral position compared to males. The number of toss and turn or waking events during sleep increased with age, and older individuals went to sleep earlier and woke up earlier. Among the symptoms of fatigue, pain, discomfort with changing position, snoring, coughing and breathing discomfort, participants reported their highest levels of discomfort due to sleepiness after waking, and they experienced the least head pain. Participants who used a regular-type pillow had poorer satisfaction on multiple comfort and support factors (support, comfort, height suitability, shape suitability) compared with those who used a functional-type pillow. Less head fatigue, less neck fatigue and less shoulder pain had significant effects on sleep quality. To reduce neck fatigue and shoulder pain, designers should consider the height for neck support in the lateral position. To reduce neck fatigue, it is desirable to use materials like latex or memory foam that provide neck support, which can improve sleep quality. The findings of this study contribute to a better understanding of sleep habits and characteristics of pillow comfort and provide practical guidelines for better pillow designs.

## 1. Introduction

Sleep accounts for one-third of the human lifespan [1] and is an important activity of everyday life that allows humans to recover physically and psychologically in preparation for the next day [2,3]. The importance of sleep is increasingly emphasized in the public sphere, and the proportion of people complaining of sleep disorders is reportedly increasing [4]. Low-quality sleep is related to less sleep, pain during sleep and breathing problems [5,6,7].

Previous studies have investigated sleep duration, bedtime and wake-up time of Koreans [8,9,10,11,12,13,14,15,16]. As interest in sleep has increased, so has interest in pillows for restful sleep. Various pillow types have been released into the market with different sizes, heights and materials. The use of proper sleeping accessories is an important way to improve sleep quality. For example, a pillow supports the head during sleep, whereas improper pillow support can cause neck stiffness, physical fatigue and pain, which can reduce sleep quality. Therefore, it is necessary to have a general understanding of the factors that can affect the quality of sleep, such as sleep habits and sleeping symptoms associated with pillow comfort and support.

Previous studies have been conducted on pillow design elements (materials, shape, contour), and pain caused by various pillow types [17,18,19,20,21,22,23,24,25,26,27]. Gordon and Grimmer-Somers [21] reported that latex materials can reduce headache and shoulder pain. In patients with chronic neck pain, a pillow that maintained the correct cervical curve of the neck could reduce neck pain and headaches [23]. In a study of pillow design elements, Kushida et al. [24] reported that cervical pillows decreased abnormal breathing events per hour during sleep in patients with sleep apnea. Cai and Chen [25] analyzed participant’s actual sleeping positions and suggested that a pillow with a high side height could improve sleep quality. Ren et al. [26] studied the importance of proper pillow height; as the height of a pillow increased, the average and highest pressure on the head and cervical spine also increased. Liu et al. [27] proposed a new pillow design with a proper combination of head, neck and shoulder support to improve sleep quality.

However, there are many considerations that have not been addressed in previous studies. Few studies have analyzed the relationship between pillow-related factors and sleep habits. In particular, previous studies did not sufficiently consider the different pillow design factors on sleeping symptoms. Previous research has been conducted by selecting a specific pillow and testing its overall shape, often by comparing a regular pillow with a cervical pillow. Shields et al. [17] claimed that there was insufficient evidence that cervical pillows reduced chronic neck pain compared with regular pillows. Therefore, it is necessary to examine pillow design factors, such as shape and materials, to understand how they impact sleep and sleeping symptoms. 

The purpose of this study is to investigate the sleep habits and sleeping symptoms relating to the comfort and support characteristics of pillows through a survey in Korean adults, and to analyze any differences by age, sex, sleeping position or pillow type. 

## 2. Methods

### 2.1. Study Design

Survey data for this study, using convenience sampling, were collected online and offline for four months (from 1 August to 30 November 2017) after deliberation by the Institutional Review Board of Kyung Hee University (KHSIRB-17-023). Most of the participants lived in a metropolitan area in Korea and comprised students studying in Kyung Hee University (most in their 20s), office workers and housewives (other ages). Only healthy participants were included in the study, as per a questionnaire (aged 20 to 76 years, Body Mass Index (BMI) 17.1 to 40.6 kg·m^−2^). The total number of participants was 332, and six people were excluded from the analysis because they did not complete the questionnaire or did not respond to the pillow material-type questions. Participants were asked to respond to a questionnaire (Table 1) that consisted of 32 questions designed to assess the sleep habits, sleeping symptoms, comfort and support characteristics of pillows, as well as perceptions of pillow suitability. We developed the questions about sleep habits (average sleep latency, bedtime, wake-up time, sleep duration, number of tosses and turns or waking events and sleeping position during the preceding month) and symptoms (snoring or coughing, breathing and sleepiness during waking hours) based on the Korean version of the Pittsburgh Sleep Quality Index (PSQI-K) and questions about pillow-related factors (support, comfort, fatigue, height and shape) from existing pillow studies [17,18,19,20,21,22,23,24,25,26,27]. 

### 2.2. Sleep Habits and Sleeping Symptoms

To develop survey questions for this study, the factors related to sleep and representative methods were examined to evaluate subjective sleep habits and sleeping symptoms relating to the comfort and support characteristics of pillows. Common questions were extracted to understand the sleep habits of healthy adults. Pilcher et al. [5] reported that indicators that affected sleep included quantitative factors such as sleep duration, bedtime, wake-up time and number of waking events during sleep, as well as qualitative factors such as self-reported quality of sleep or satisfaction with sleep. Common factors assessed by researchers included sleep duration, sleep quality, bedtime and sleep latency [28,29,30,31,32]. Methods for subjectively evaluating sleep quality were used, including the Verran and Snyder-Halpern Sleep Scale [28], the Pittsburgh Sleep Quality Index (PSQI) [33] and the Karolinska Sleep Diary [34]. Among those, the PSQI is the most commonly used measure [35,36,37], and it measures sleep quality and sleep disturbance during the preceding month. Our sleep habit questions were based on the PSQI-K which has been tested for its reliability and validity [38]. In addition, some questions about subjective assessment were added using a Likert scale (see Table 1 for the entire questionnaire). The Likert scale for the overall sleep quality was answered using the following criteria: for sleep quality, with 1 being low sleep quality and 7 high sleep quality; for sleepiness during the day, with 1 being discomfort and 7 comfort; and for the number and degree of severity of sleep-related symptoms with 1 = strong negative, 2 = negative, 3 = somewhat negative, 4 = neutral, 5 = somewhat positive, 6 = positive and 7 = strong positive. The question of sleeping position was largely divided into supine and lateral positions. The supine position was defined as “Mainly sleep in a supine position” and “Mainly sleep in a supine position but also sleep in a lateral position”. The lateral position was defined as “Mainly sleep in a lateral position” and “Mainly sleep in a lateral position but also sleep in a supine position”.

### 2.3. Pillow Types 

Market research and a related literature review were conducted to develop questions about the comfort and support characteristics associated with pillow use. Previous studies about pillows were systematically reviewed and information about more than 200 commercially available pillows was collected and classified into six types according to shape and contour (Table 2). According to the studies of Yokura et al. [39] and Hur and Yang [22], design factors of pillow type such as height shape, and subjective factors of pillow comfort and fatigue could affect pillow usability. In this study, we selected support, height and shape as pillow design factors. Since a pillow affects the head, neck and shoulder comfort, questions directed to these body parts in terms of comfort, fatigue, pain and overall satisfaction were examined. 

### 2.4. Analysis

The collected data were analyzed using IBM SPSS 23 statistical software. A cluster analysis was performed to divide the participants into three groups (high, medium, low) according to height and BMI. In the case of BMI groups, the WHO Asia-Pacific guideline of BMI was used to classify the degree of obesity [40], with “underweight” being less than 18.5 kg·m^−2^, “normal weight” being 18.5–22.9 kg·m^−2^, “overweight” being 23–24.9 kg·m^−2^ and “obese” being more than 25 kg·m^−2^. One-way ANOVA was performed to analyze differences of sleep habits, sleeping symptoms and the comfort and support characteristics of pillows according to age, height, BMI and pillow types. As a post-hoc analysis, Fisher’s least significant difference (LSD) test was used to identify differences between pillow-use groups. Independent *t*-tests were performed to analyze differences according to sex and sleeping position. In addition, the scores of each variable were compared by statistical analysis based on means and standard deviation. To explain the relationship between symptoms during sleep and after waking and quality of sleep, a multiple linear regression analysis was performed. The *p*-values less than 0.05 were considered statistically significant.

## 3. Results

Three hundred and thirty-two adults aged 20–76 years responded to our study survey. The mean age of participants was 41.6 years (male: Mean = 40.4 years, SD = 15.2 years; female: Mean = 42.9 years, SD = 15.4 years). Participants were classified into three age groups: 178 (54%) people in their 20s–30s, 81 (24%) in their 40s–50s and 73 (22%) in their 60s–70s. There were 176 (53%) male and 156 (47%) female participants. Height was classified into three groups: 107 (33%) people with a short stature (less than 162.5 cm), 134 (40%) with a medium stature (162.5–174 cm) and 91 (26%) with a tall stature (more than 174 cm). Body Mass Index (BMI) was also classified into three groups: 142 (43%) people with a low BMI (less than 22.4 kg·m^−2^), 143 (43%) with a medium BMI (22.4–26.7 kg·m^−2^) and 47 (14%) with a high BMI (more than 26.7 kg·m^−2^).

### 3.1. Sleep Habits

Descriptive statistics regarding the sleep habits and sleeping symptoms of participants are presented in Table 3. There were significant differences in the number of tossing and turning or waking events, wake-up times and sleep durations among age groups (*p* < 0.05; Table 3). The number of tossing and turning or waking events during sleep increased with age.

There were significant differences in bedtime according to sex (*p* < 0.05; Table 3). Females went to bed earlier than males. Older individuals went to sleep earlier and woke up earlier. Sleep duration was shortest for participants in their 40s–50s. Subjective sleep evaluation indicated that the older an individual was, the more snoring or coughing symptoms and greater discomfort with breathing they experienced during sleep. Many participants reported feeling sleepy during waking hours. Head pain after waking was not a common complaint, but older participants experienced more head pain than younger individuals. 

Sleep habits differed significantly according to height and BMI groups (*p* < 0.05; Table 3). The tallest group had the lowest number of tossing and turning or waking events and the earliest bedtime and wake-up time. The tallest participants felt the least head pain after sleeping (high score). The overall sleep quality was also the highest for the tallest individuals. There were no significant differences in sleeping position according to height. The medium BMI class showed the highest number of tossing and turning or waking events, and the highest BMI group had the shortest sleep duration. In addition, individuals in the high BMI class had poorer scores for snoring or coughing symptoms and discomfort with breathing during sleep than medium and low BMI classes. There were no significant differences in sleeping position by BMI.

The most common sleeping position was “Mainly sleep in a supine position but also sleep in a lateral position” (44%), with 9% of participants reporting sleeping mainly in a supine position (Table 4). Movement from a supine to a lateral position or vice versa was more common than a fixed sleeping position. Overall, a supine position was more common than a lateral position. Although most males and females slept mainly in a supine position, females slept more often on their backs or sides than males, and males slept more often in a supine position. 

### 3.2. Comfort and Support Characteristics of Pillows

Most participants used regular-type pillows (54%), but 22% of participants used contour-type pillows (peanut-shaped pillow: 8%; functional A-type pillow: 7%; functional B-type pillow: 8%; other: 2%). The score for the pillow in use was lowest for shoulder support, followed by shoulder fatigue (Table 5). The support and comfort questions scored higher on the head than the shoulders and neck. There were significant differences in shoulder support, neck fatigue and pillow temperature satisfaction among the age groups (*p* < 0.05; Table 5). Participants in their 20s–30s reported lower scores for shoulder support (tended to feel the least support) and neck fatigue (tended to feel the least fatigue) than the other two age groups. A low satisfaction score was reported for the older group. Neck support and shape suitability of the pillow for the neck and shoulder differed significantly by sex. Females had lower scores than males for these questions, indicating less satisfaction with neck support, shape suitability and shoulder support of their pillows. There were also significant differences in head support, head fatigue and shape suitability of the head and neck support according to sleeping position (*p* < 0.05; Table 5). Participants who slept in a lateral position had lower scores for these items (i.e., received less support and experienced more fatigue and less shape suitability) than those who slept in a supine position.

Support, comfort and shape suitability of neck and shoulder, head support and height suitability of neck differed significantly by pillow types (*p* < 0.05) according to the ANOVA results of comfort and support factors by pillow types (Table 6). Participants who used functional-type pillows reported greater head, neck and shoulder support and neck comfort than those who used other pillow types. Participants who used a peanut-shaped pillow had the highest shoulder comfort. Participants who used contour-type pillows and functional A-type pillows had the highest scores for neck height suitability. The functional A-type pillows received the highest shape-suitability score for the neck, whereas the peanut-shaped pillows received the highest shape-suitability score for the shoulders. Regular-type pillows scored the lowest on several questions.

Table 7 shows the results of multiple linear regression analysis. Less fatigue of the head and neck, less shoulder pain and comfort when changing position during sleep have significant effects on quality of sleep. 

Table 8 shows the results of multiple linear regression analyses of sleeping positions (supine and lateral) identifying the relationship between pillow design factors and major sleeping symptoms that have significant effects on the sleep quality. The stepwise method was used to select independent variables for the regression analyses. As shown in Table 8, the significant pillow factors for major sleeping symptoms were slightly different according to sleeping positions. Overall, sleeping symptoms were significantly impacted by the corresponding pillow factors except for the shoulder area. Also, the shape factors were significant for every major symptom, but the height suitability factors were significant only for the lateral position.

**Figure 1:**
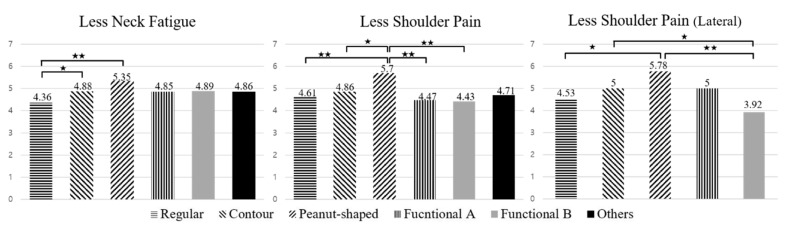
Post-hoc test results using Fisher’s least significant difference (LSD) method for major sleeping symptoms by pillow types (★★: *p* < 0.01, ★: *p* < 0.05).

Figure 1 shows the LSD post-hoc results of major sleeping symptoms that were significantly different by pillow type. There were significant differences in neck fatigue (F (5320) = 3.223; *p*-value = 0.007) and shoulder pain (F (5320) = 2.536; *p*-value = 0.029) according to pillow type. The results of the ANOVA between the major sleeping symptoms and pillow types according to sleeping position showed no significant difference for the supine position. Otherwise, those sleeping in the lateral position reported significantly less shoulder pain (F (4138) = 2.688; *p*-value = 0.034). The results of post-hoc analysis using Fisher’s LSD test showed that regular-type pillows scored lower than other pillow types, and peanut-shaped pillows and contour-type pillows scored higher for less symptoms than other pillow types. Overall, functional type pillows scored lower for shoulder pain. In particular, in the lateral position, the lowest scores were found in the functional B-type pillows, and the scores were significantly different from the functional A-type pillows. 

Regarding pillow materials (feathers, cotton, plastic capsules, memory foam, latex and natural material), pillows made of cotton had the highest proportion of users (37%; 121 out of 326), followed by latex and memory foam (23%; 75 out of 326), feathers (7%; 22 out of 326), natural materials (6%; 21 out of 326) and plastic capsules (4%; 13 out of 326). Neck fatigue differed significantly by material, with plastic capsule (Mean = 5, SD = 1.29), memory foam (Mean = 4.92, SD = 1.47) and latex materials (Mean = 4.95, SD = 1.28) showing less neck fatigue than other materials.

## 4. Discussion

Deyonker et al. [41] reported that the average sleep duration of healthy adults is about 7 h, and that most adults sleep for at least 6.5 h. The Korean National Center for Health Statistics reported in 1977 that most people sleep for more than 7 h, but less than 8 h [42]. In the current study, the average sleep duration of Korean adults was 6 h 48 min, which is less than the average sleep duration of 7 h 2 min reported for British adults (mean = 7.04 h, SD = 1.55 h) [43], and less than the recommended sleep duration of 7–8 h [16,44]. The overall sleep quality for our participants was 4.84 points and considered slightly poor after being assessed on a seven-point scale (1 (low sleep quality) to 7 (high sleep quality)). This finding was similar to that reported by Keklund [45] for sleep quality of American adults (3.4 points on a five-point scale). The average bedtime was 12:06 a.m., and the average wake-up time was 6:54 a.m. American adults previously reported going to bed at 11:28 p.m. on average [46]. This difference could be due to the high ratio of younger to older people in the current study. In addition, the score for sleepiness during waking hours was lower (4.4 points), indicating some degree of sleepiness during the day (1 (discomfort) to 7 (comfort)), which suggests that daytime sleepiness is perceived as a greater discomfort than other related symptoms. Young people experienced more daytime sleepiness than middle-aged or elderly people, likely due to the excessive use of smartphones and digital media, which can interfere with sleep and cause fatigue during the day [47,48,49,50]. The proportion of young people (20s–30s) in our study population was more than 50%, which could account for some degree of sleepiness reported during waking hours. 

Most participants reported sleeping mainly in a supine position with some time in a lateral position, followed by participants who mainly slept in a lateral position with some time in a supine position. This may be to avoid blood stasis when maintaining the same sleeping position for too long [51]. The percentage of individuals who slept mostly or entirely in a supine position (52%) was slightly higher than that of individuals who slept mostly or entirely in a lateral position (44%). This is in contrast to a study of Park and Lee [11], who reported that a lateral position was most common for Korean adults. According to the results of this study, participants who usually slept in a lateral position were more unsatisfied with head support and head shape suitability of the pillow and experienced less neck support and more neck fatigue than those who slept in a supine position. In other words, the pillows the participants were using did not seem to be offering adequate support for those who slept in a lateral position. Sleeping position differed significantly by sex. The ratio of “Mainly sleeping in a supine position but also sleeping in a lateral position” was high in both males and females (males: 50%; females: 39%), and the ratio of “Mainly sleeping in a supine position” was low (males: 12%; females: 5%). However, when the sleeping position was divided into supine and lateral positions, the percentage of females who slept on their sides was higher than the percentage of males who slept on their sides (male: 40%; female: 53%), which is consistent with the findings of Choi et al. [52]. 

Participants reported tossing and turning or waking twice a night on average. This is slightly more than the 1.5 times reported by Choi et al. [53] for 1000 residents of Seoul. Among the statistically significant data observed by age groups, the natural aging phenomenon may explain the increases in the number of tossing and turning or waking events, earlier bedtime and wake-up time, breathing discomfort, snoring and coughing during sleep and morning headaches experienced among the older groups [54,55]. Unlike the results of this study, there were some studies that showed no significant difference by age [55], but our results were consistent with the preponderances of studies that reported a difference in those overall sleep habits and symptoms by age groups [52,56,57]. 

There was a significant difference between age groups in neck fatigue and shoulder support and temperature suitability of the pillow. Younger participants reported more neck and shoulder fatigue than older participants. Pain in the neck area after sleep may be due to poor posture during everyday tasks when in a static position for long periods [11], which could be why individuals in their 20s and 30s experienced more neck pain, as they spend a large amount of time staring at computer or smartphone screens. For shoulder support, older participants can overcome neck and shoulder discomfort by paying attention to physical health and investing in products that can improve neck and shoulder health.

Regarding sleep quality and symptoms, pillow shape was the main factor causing the major sleeping symptoms (head fatigue, neck fatigue and shoulder pain) that affected sleep quality. Therefore, it could be concluded that proper shape design for each part (head, neck and shoulders) is the most important factor of an effective pillow for optimal comfort. Also, according to Figure 1, overall scores were low for regular-type pillows and high for peanut-shaped and contour-type pillows, implying that pillows with a flat horizontal plane and gentle vertical curve for the neck and head area could be an effective shape for pillows. In cases of functional type pillows with a large height difference between the side and center area, suitability scores were different according to type of pillow. Therefore, the effectiveness of these pillow types requires further study for the validation of their effectiveness and comfort. 

According to the results of our study, effective pillow characteristics are different according to sleeping position. Overall, neck shape suitability had a significant effect on head fatigue in the supine position, unlike the lateral position. This could be attributed to the difference in supporting areas between sleeping positions. When sleeping in a supine position, both the head and neck areas of a pillow support their respective area of body, but less neck support in a lateral position. For neck fatigue, height suitability was additionally significant for the lateral position. This may mean that the pillows available on the market were not properly considered for their height according to a lateral position. For shoulder pain, the significant variables were similar for different sleeping positions, but the neck-height suitability was higher for the lateral position than the supine position. This means that shoulder discomfort may be increased as a result of improper neck height in the lateral position. Therefore, there is a need to identify the adequate neck height for those who sleep mainly in a lateral position in order to reduce neck fatigue and shoulder pain.

Neck fatigue differed significantly between pillow materials and symptoms related to sleep quality. Neck fatigue was rarer among users of pillows filled with plastic capsules, latex or memory foam than among those using pillows filled with feathers or cotton. Plastic capsules, latex and memory foam are more elastic and less fluffy than feathers and cotton, allowing them to maintain their shape better and possibly offer better neck support. Therefore, a lower level of fluffiness and a higher degree of elasticity are expected to help relieve neck fatigue. Thus, to reduce sleep discomfort for a user, a pillow that supports the neck with a material such as latex or memory foam is appropriate.

This study has the following limitations. First, many of the participants in this study were university students and young adults working at Kyung Hee University, and fewer middle-aged and elderly people responded than younger people. Therefore, the reported results of this study could be biased toward the younger population. Although more than 300 people responded, the results cannot be generalized to all Korean adults. In addition, this study did not consider factors such as occupation, smoking status, coffee intake and other physical or mental illnesses that could have an impact on sleep. There were also some limitations to the subjective sleep quality questionnaire. We asked about overall sleep habits and symptoms related to sleep quality, but we did not conduct objective assessments, (e.g., estimation of sleep using actigraphs). It was also not possible to compare the sleep data directly with the PSQI, since the overall quality of sleep score in this study was derived from questions from a few questionnaires, although mostly based off the PSQI-K. In addition, we did not include the ear, an important body part with respect to comfort and pillow use when sleeping, in this study. Thus, there is a need for further research, as pain may develop with an ear leaning on the pillow. In terms of pillow shape and size, since our study did not measure the actual size of the pillows and did not collect the detailed pillow shapes, we could not provide detailed pillow design guidelines. Even in pillow materials, the plastic capsules and feathers were 6.7% and 4.0% of the total pillows, respectively, compared to 37.1% for cotton and 23% for memory foam. The balance of the number of experimental pillows remains a limitation of this study. Attention should also be paid to other pillow characteristics such as hardness and thermal conductivity. Furthermore, sleep quality can be influenced not only by pillow comfort but also by mattresses and individual lifestyle factors. Therefore, future research should be conducted to balance the sex and age of the participants, consider a variety of external variables and clarify the differences among pillow design to unravel the detailed relationship between sleep quality and pillow design factors.

## 5. Conclusions

This study investigated sleep habits, sleeping symptoms and the comfort and support characteristics of pillows, and analyzed their relationships with each other. The overall quality score of sleep for Korean adults was on a similar level to other countries. Our participants, on average, reported a later bedtime, shorter sleep duration, more tossing and turning and waking and more daytime sleepiness than previous studies. This study reported frequent changes in sleeping positions, and the supine position was the preferred sleeping position. The sleep-related discomforts that negatively affected sleep quality were head and neck fatigue, shoulder pain and postural change during sleep. In order to reduce the negative symptoms of the head, neck and shoulders, it is important to properly design a pillow’s shape. In cases of neck fatigue, it is desirable to design a pillow using materials such as latex or memory foam that have good neck support. In addition, to reduce neck fatigue and shoulder pain, it is necessary to consider the proper height of the neck support area for the lateral position. The findings of this study contribute to a better understanding of pillow comfort and support and their potential impact on sleep quality, suggesting the importance of pillow designs.

## Figures and Tables

**Table 1:** Questionnaire to assess sleep habits, sleeping symptoms and the comfort and support characteristics of pillows.

Questions	Variables	Method
How many times do you toss and turn or wake in the night or early morning?	Toss and turn or waking (number)	Short answer
How long does it usually take you to fall asleep?	Sleep latency (minutes)	
When do you usually go to bed at night?	Bedtime (time ^b^)	
When do you usually get up in the morning?	Wake-up time (time ^b^)	
How many hours of actual sleep do you get at night?	Sleep duration (hours)	
What type of pillow do you currently use? ^a^	Pillow type	Multiple choices
What is your usual sleeping position?	Sleeping position	
Do you feel comfortable when changing position during sleep?	Comfort position changes	7-point Likert scale
Do you feel less discomfort of snoring or coughing during sleep?	Less snoring or coughing	
Are you comfortable with breathing during sleep?	Breathing	
Do you have less pain in your head after waking?	Less head pain	
Do you have less pain in your neck after waking?	Less neck pain	
Do you have less pain in your shoulders after waking?	Less shoulder pain	
Do you feel less sleepy during your waking hours?	Less sleepiness during waking hours	
How would you rate your sleep quality overall?	Sleep quality	
Does your pillow support your head well?	Head support	
Does your pillow support your neck well?	Neck support	
Does your pillow support your shoulder well?	Shoulder support	
Is the head support of your pillow comfortable?	Head comfort	
Is the neck support of your pillow comfortable?	Neck comfort	
Is the shoulder support of your pillow comfortable?	Shoulder comfort	
Do you feel less fatigue from the head support of your pillow?	Less head fatigue	
Do you feel less fatigue from the neck support of your pillow?	Less neck fatigue	
Do you feel less fatigue from the shoulder support of your pillow?	Less shoulder fatigue	
Is the temperature of your pillow suitable?	Temperature suitability	
Is the head height of your pillow suitable?	Head height suitability	
Is the neck height of your pillow suitable?	Neck height suitability	
Is the head shape of your pillow suitable?	Head shape suitability	
Is the neck shape of your pillow suitable?	Neck shape suitability	
Is the shoulder shape of your pillow suitable?	Shoulder shape suitability	
Are you satisfied with your pillow?	Overall satisfaction	
Would you be willing to repurchase your pillow?	Willing to repurchase	

^a^ Detailed descriptions of pillow types are provided in Table 2; ^b^ Time is expressed using a 24-h clock.

**Table 2:** Classification and definition of pillow types.

Pillow Type	Definition	Example
Regular-type pillow	- Typical oval or rectangular shape	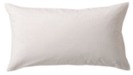
Contour-type pillow	- Different heights for head and neck support	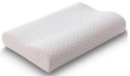
Peanut-shaped pillow	- Different heights for head and neck support - U-shaped to surround the neck and shoulders	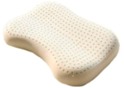
Functional A-type pillow	- Different heights for head and neck support - Neck supported by a neck support	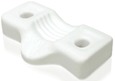
Functional B-type pillow	- Different heights for head and neck support - Neck surrounded by a neck support	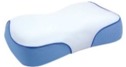
Other	- Wooden pillow, etc.	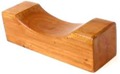

**Table 3:** *T-test* and ANOVA results for sleep habits (Mean (SD)).

Sleep Habits		Total	Age (Years)			Sex		Height			BMI		
			20–39	40–59	60–76	M	F	S	M	T	L	M	H
Sleep habits	Toss and turn or waking up (number)	2.0 (1.1)	1.6 * (0.9)	2.3 * (0.7)	2.7 * (1.1)	2.0 (1.1)	2.1 (2.1)	2.3 * (1.1)	2.0 * (1.1)	1.7 * (0.9)	1.9 * (1.0)	2.2 * (1.1)	2.0 * (1.0)
	Sleep latency (minutes)	28.6 (25.0)	30.1 (21.5)	25.9 (14.2)	28.1 (29.3)	27.4 (19.1)	29.9 (25)	30.5 (26.6)	27.1 (18.7)	28.6 (20.7)	30.6 (26.8)	28.0 (18.7)	24.4 (14.1)
	Bedtime (time)	24.1 (1.4)	24.8 * (1.5)	23.6 * (1.1)	22.9 *(1.1)	24.2 * (1.7)	23.9 * (1.4)	23.7 * (1.4)	24.0 * (1.5)	24.8 * (1.6)	24.2 (1.6)	24.0 (1.6)	24.2 (1.5)
	Wake-up time (time ^a^)	7.0 (1.6)	7.9 * (1.4)	6.2 * (0.9)	5.8 * (2.1)	7.1 (1.9)	6.9 (1.6)	6.5 * (1.6)	7.0 * (2.0)	7.6 * (1.5)	7.2 (1.7)	6.9 (2.0)	6.8 (1.5)
	Sleep duration (hours)	6.8 (1.1)	7.0 * (1.2)	6.6 * (1.0)	6.7 * (1.1)	6.7 (1.2)	6.9 (1.1)	6.8 (1.0)	6.8 (1.2)	6.8 (1.2)	7.0 * (1.2)	6.7 * (1.1)	6.6 * (1.0)
Symptoms during sleep	Comfort Position changes	4.9 (1.5)	4.9 (1.5)	4.8 (1.3)	4.7 (1.3)	4.9 (1.4)	4.8 (1.5)	4.8 (1.4)	4.8 (1.5)	5.1 (1.3)	4.8 (1.5)	5.0 (1.3)	4.7 (1.3)
	Less Snoring or coughing	4.8 (1.5)	5.1 * (1.6)	4.5 * (1.6)	4.6 * (1.5)	4.6 (1.6)	5.1 (1.5)	4.9 (1.6)	4.8 (1.6)	4.8 (1.6)	5.2 * (1.6)	4.8 * (1.5)	4.1 * (1.6)
	Breathing	5.3 (1.4)	5.6 * (1.3)	5.1 * (1.4)	4.9 * (1.5)	5.2 (1.4)	5.4 (1.4)	5.4 (1.3)	5.2 (1.5)	5.4 (1.4)	5.4 * (1.4)	5.3 * (1.4)	4.8 * (1.4)
Symptoms after waking	Less Head pain	5.4 (1.3)	5.5 * (1.3)	5.3 * (1.2)	5.1 * (1.3)	5.5 (1.3)	5.3 (1.2)	5.2 * (1.3)	5.4 * (1.3)	5.6 * (1.2)	5.4 (1.2)	5.3 (1.3)	5.4 (1.3)
	Less Neck pain	4.9 (1.4)	4.8 (1.6)	5.0 (1.2)	5.0 (1.4)	5.0 (1.5)	4.7 (1.4)	4.7 (1.4)	5.0 (1.4)	5.0 (1.6)	4.9 (1.4)	4.8 (1.4)	5.0 (1.4)
	Less Shoulder pain	4.7 (1.5)	4.7 (1.6)	4.7 (1.2)	4.7 (1.4)	4.8 (1.4)	4.5 (1.5)	4.6 (1.5)	4.6 (1.5)	4.9 (1.4)	4.7 (1.5)	4.6 (1.5)	4.9 (1.4)
	Less Sleepiness during waking hours	4.4 (1.4)	4.4 (1.5)	4.4 (1.4)	4.5 (1.3)	4.4 (1.4)	4.5 (1.4)	4.5 (1.3)	4.3 (1.4)	4.6 (1.5)	4.6 (1.5)	4.3 (1.4)	4.5 (1.4)
Less Fatigue	Head	5.1 (1.2)	5.2 (1.3)	5.0 (1.1)	5.0 (1.2)	5.1 (1.2)	5.1 (1.2)	5.0 (1.2)	5.1 (1.2)	5.2 (1.1)	5.0 (1.3)	5.2 (1.1)	5.0 (1.0)
	Neck	4.6 (1.4)	4.4 * (1.5)	4.9 * (1.2)	4.9 * (1.2)	4.7 (1.4)	4.5 (1.4)	4.6 (1.4)	4.6 (1.4)	4.6 (1.5)	4.5 (1.4)	4.7 (1.5)	4.6 (1.3)
	Shoulder	4.2 (1.4)	4.1 * (1.5)	4.4 * (1.3)	4.5 * (1.4)	4.3 (1.5)	4.2 (1.4)	4.3 (1.4)	4.2 (1.5)	4.2 (1.4)	4.3 (1.4)	4.3 (1.5)	4.2 (1.3)
Sleep quality		4.8 (1.3)	4.9 (1.3)	4.8 (1.3)	4.7 (1.2)	4.9 (1.3)	4.8 (1.3)	4.7 * (1.2)	4.8 * (1.3)	5.1 * (1.2)	4.9 (1.3)	4.9 (1.3)	4.6 (1.2)

* Analysis of variance (age, height, BMI) and independent *t*-test value (sex), *p* ≤ 0.05; ^a^ Time is expressed using a 24-h clock.

**Table 4:** Descriptive statistics of sleeping position.

Position	*n*	Detailed Descriptions of Position	*n*	Male	Female
Supine	174 (52%)	Mainly sleep in a supine position	29 (9%)	21 (72%)	8 (28%)
		Mainly sleep in a supine position but also sleep in a lateral position	145 (44%)	84 (58%)	61 (42%)
				105 (60%)	69 (47%)
Lateral	146 (44%)	Mainly sleep in a lateral position	58 (18%)	31 (53%)	27 (47%)
		Mainly sleep in a lateral position but also sleep in a supine position	88 (27%)	38 (43%)	50 (57%)
				69 (40%)	77 (53%)
Other	12 (4%)	Prone position, etc.	12 (4%)	2 (17%)	10 (83%)
		Total	332	176	156

**Table 5:** *T-test* and ANOVA results for comfort and support characteristics of pillows (Mean (S.D.)).

Variables		Total	Age (Years)			Sex		Sleeping Position	
			20–39	40–59	60–76	Male	Female	Supine	Lateral
Support	Head	5.1 (1.2)	5.2 (1.3)	5.2 (1.1)	5.0 (1.2)	5.1 (1.3)	5.1 (1.2)	5.3 * (1.1)	4.9 * (1.3)
	Neck	4.7 (1.4)	4.6 (1.5)	4.9 (1.2)	4.9 (1.3)	4.6 * (1.5)	4.9 * (1.3)	4.9 * (1.4)	4.5 * (1.4)
	Shoulder	3.8 (1.5)	3.6 * (1.5)	4.1 * (1.4)	4.1 * (1.4)	3.7 (1.5)	3.9 (1.5)	3.9 (1.5)	3.7 (1.5)
Comfort	Head	5.2 (1.2)	5.2 (1.3)	5.3 (1.1)	5.0 (1.2)	5.2 (1.2)	5.2 (1.3)	5.2 (1.3)	5.2 (1.2)
	Neck	4.7 (1.4)	4.6 (1.5)	5.0 (1.2)	4.8 (1.4)	4.7 (1.5)	4.7 (1.3)	4.9 (1.4)	4.6 (1.3)
	Shoulder	4.1 (1.5)	3.9 (1.5)	4.3 (1.4)	4.1 (1.4)	4.0 (1.5)	4.2 (1.4)	4.2 (1.5)	3.9 (1.4)
Less Fatigue	Head	5.1 (1.2)	5.2 (1.3)	5.0 (1.1)	5.0 (1.2)	5.1 (1.2)	5.1 (1.2)	5.2 (1.2)	5 (1.2)
	Neck	4.6 (1.4)	4.4 * (1.5)	4.9 * (1.2)	4.9 * (1.2)	4.7 (1.4)	4.5 (1.4)	4.8 * (1.4)	4.4* (1.5)
	Shoulder	4.2 (1.4)	4.1 * (1.5)	4.4 * (1.3)	4.5 * (1.4)	4.3 (1.5)	4.2 (1.4)	4.4 (1.5)	4.1 (1.4)
Height suitability	Head	5.0 (1.3)	5.0 (1.4)	5.0 (1.3)	5.1 (1.3)	5.0 (1.3)	5.0 (1.3)	5.1 (1.3)	4.8 (1.3)
	Neck	4.7 (1.4)	4.6 (1.4)	4.7 (1.4)	4.9 (1.3)	4.7 (1.5)	4.7 (1.3)	4.8 (1.4)	4.5 (1.4)
Shape suitability	Head	5.1 (1.3)	5.1 (1.3)	5.1 (1.2)	5.0 (1.2)	5.1 (1.2)	5.0 (1.3)	5.2* (1.2)	4.9* (1.3)
	Neck	4.8 (1.4)	4.7 (1.4)	4.9 (1.3)	4.9 (1.3)	4.7 * (1.4)	4.8 * (1.2)	4.8 (1.3)	4.7 (1.4)
	Shoulder	4.4 (1.4)	4.4 (1.5)	4.5 (1.4)	4.4 (1.2)	4.3 * (1.5)	4.4 * (1.3)	4.5 (1.3)	4.3 (1.5)
Temperature suitability		5.0 (1.3)	5.2 * (1.2)	4.8 * (1.3)	4.7 * (1.4)	5.0 (1.3)	5.0 (1.3)	5.1 (1.4)	4.9 (1.4)
Overall satisfaction		4.9 (1.3)	4.9 (1.4)	4.8 (1.2)	4.9 (1.2)	4.9 (1.3)	4.8 (1.3)	5.0 (1.2)	4.8 (1.4)
Willingness to repurchase		4.6 (1.6)	4.4 (1.6)	4.8 (1.5)	4.7 (1.6)	4.5 (1.6)	4.6 (1.6)	4.7 (1.6)	4.4 (1.6)

* Analysis of variance (age), independent *t*-test value (sex, sleeping position), *p* ≤ 0.05.

**Table 6:** ANOVA results of comfort and support by pillow types (Mean (SD)).

Variables		Regular Pillow	Contour Pillow	Peanut-Shaped Pillow	Functional A Pillow	Functional B Pillow	Other
Support	Head	4.9 * (1.3)	5.3 * (1.1)	5.2 * (1.2)	5.6 * (1.0)	5.5 * (1.2)	6.0 * (0.6)
	Neck	4.3 * (1.5)	5.1 * (1.1)	5.2 * (1.0)	5.5 * (1.1)	5.5 * (0.9)	5.3 * (1.1)
	Shoulder	3.5 * (1.5)	4.2 * (1.3)	4.1 * (1.6)	4.1 * (1.3)	4.7 * (1.4)	3.6 * (1.6)
Comfort	Head	5.1 (1.3)	5.3 (1.1)	5.4 (1.1)	5.5 (0.9)	5.2 (1.4)	4.9 (1.1)
	Neck	4.4 * (1.5)	5.0 * (1.1)	5.0 * (1.3)	5.3 * (1.1)	5.3 * (1.4)	4.3 * (1.1)
	Shoulder	3.7 * (1.5)	4.4 * (1.3)	4.8 * (1.1)	4.2 * (1.4)	4.7 * (1.5)	3.7 * (1.1)
Height suitability	Head	4.9 (1.3)	5.1 (1.3)	4.8 (1.5)	5.5 (1.1)	5.0 (1.4)	5.1 (1.1)
	Neck	4.4 * (1.4)	5.1 * (1.3)	4.6 * (1.6)	5.1 * (1.3)	4.9 * (1.4)	5.0 * (1.4)
Shape suitability	Head	4.9 (1.3)	5.3 (1.0)	5.2 (1.5)	5.6 (1.1)	5.0 (1.4)	4.7 (1.8)
	Neck	4.4 * (1.3)	5.1 * (1.3)	5.2 * (1.3)	5.6 * (0.9)	5.1 * (1.1)	4.6 * (1.6)
	Shoulder	4.1 * (1.4)	4.6 * (1.4)	5.0 * (1.5)	4.8 * (1.2)	4.7 * (1.3)	4.4 * (1.4)
Temperature suitability		4.9 (1.3)	5.1 (1.3)	5.3 (1.3)	5.3 (1.0)	4.9 (1.2)	5.1 (1.7)
Overall satisfaction		4.7 (1.3)	5.0 (1.2)	5.1 (1.3)	5.1 (1.2)	5.1 (1.5)	4.7 (1.0)
Willingness to repurchase		4.3 (1.7)	4.8 (1.4)	4.8 (1.7)	5.0 (1.5)	5.0 (1.7)	4.4 (1.3)

* Analysis of variance, *p* ≤ 0.05.

**Table 7:** Results of regression analysis between sleeping symptoms and sleep quality.

Dependent Variable	Independent Variables: Sleeping Symptom		B	β	t	*p*-Value
Sleep quality	Less Fatigue	Head	0.243	0.225	4.573	0.000
		Neck	0.150	0.164	2.626	0.009
		Shoulder	0.094	0.104	1.816	0.070
	Less Pain	Head	0.104	0.103	1.872	0.062
		Neck	0.110	0.122	1.795	0.074
		Shoulder	0.139	0.158	2.631	0.009
	Comfort changing position		0.082	0.090	1.991	0.047
	Less Snoring or coughing		0.023	0.029	0.560	0.576
	Breathing		0.025	0.027	0.485	0.628

**Table 8:** Results of multiple linear regression analyses between major sleeping symptoms and pillow design factors according to sleeping positions.

Dependent Variables: Major Sleeping Symptoms	Sleeping Position	Independent Variables: Pillow Factors	B	β	t	*p*-Value
Less Head Fatigue	Supine	Head Support	0.382	0.372	5.738	0.000
		Head Shape	0.263	0.268	3.383	0.001
		Neck Shape	0.210	0.234	3.331	0.001
	Lateral	Head Support	0.291	0.310	3.985	0.000
		Head Shape	0.430	0.459	5.910	0.000
Less Neck Fatigue	Supine	Neck Support	0.236	0.240	3.565	0.000
		Neck Shape	0.543	0.520	7.719	0.000
	Lateral	Neck Support	0.249	0.234	2.934	0.004
		Neck Height	0.294	0.291	3.208	0.002
		Neck Shape	0.287	0.271	2.799	0.006
Less Shoulder Pain	Supine	Neck Height	0.199	0.193	2.559	0.011
		Shoulder Shape	0.487	0.447	5.941	0.000
	Lateral	Neck Height	0.296	0.275	3.371	0.001
		Shoulder Shape	0.386	0.372	4.561	0.000
